# Recent Advances in the Synthesis of Commercially Available Phosphite Antioxidants

**DOI:** 10.1002/open.202400135

**Published:** 2024-11-13

**Authors:** Yuliang Zhu, Xinyue Liu, Ying Tang, Kexin Xu, Xin Tang, Longzhi Zhu, Biquan Xiong

**Affiliations:** ^1^ Department of Chemistry and Chemical Engineering Hunan Institute of Science and Technology Yueyang 414006 P. R. China; ^2^ School of Economics and Management Hunan Institute of Science and Technology Yueyang 414006 P. R. China

**Keywords:** Antioxidants, Industrial products, Nucleophilic substitution, Phosphites, Synthesis

## Abstract

Phosphite antioxidants exhibit superior anti‐aging and color‐stabilizing properties when incorporated into polymer materials. Their synergistic antioxidative effects are particularly noteworthy when used in combination with hindered phenol antioxidants and other primary antioxidants, serving as effective secondary antioxidants, displaying noteworthy synergistic antioxidation effects. This review systematically classifies the synthetic methods for phosphite antioxidants into three distinct categories based on the types of starting materials: synthesis from phosphorus trichloride, phosphorus‐containing esters, and white phosphorus. Additionally, it delineates the reaction mechanisms associated with these approaches and provides an overview of future potential research directions and applications for organophosphorus antioxidants.

## Graphical abstract

This review categorizes synthetic methods for phosphite antioxidants based on their precursors: phosphorus trichloride, phosphorus‐containing esters, and white phosphorus. The objective is to provide a valuable reference for researchers in the field of antioxidant development, aiding in the conception of innovative antioxidants and exploring environmentally sustainable synthesis strategies that adhere to the escalating regulatory standards within industrial manufacturing.

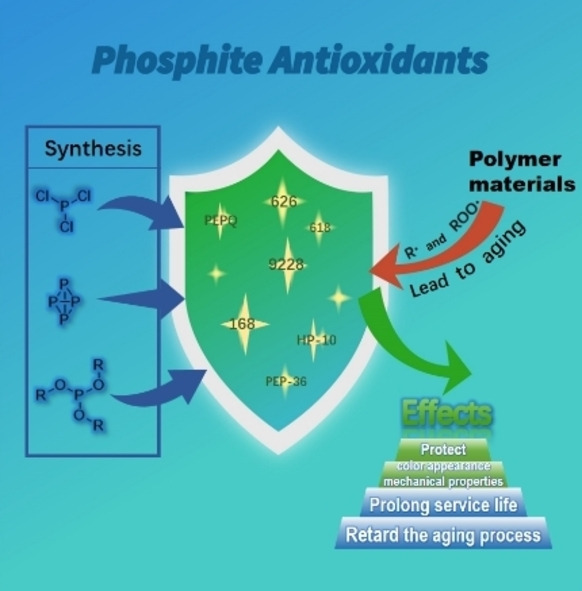

## 1 Introduction

Most of polymer materials are susceptible to the chain reactions inherent in their molecular structure,^[1]^ rendering them particularly prone to aging process, which could lead to the deterioration in color, appearance, and physical/mechanical performances,^[2]^ thereby reducing their service life of the materials and affecting their functionality. Moreover, the aging of the materials would even lead to a great waste of polymeric resources and pose risks to the environment and human safety. To mitigate these issues, antioxidants are commonly incorporated during the material processing stage.^[3]^ According to the report “Global and Chinese Antioxidant and Anticorrosion Market Status and Future Development Trends 2023–2029”, the global sales revenue for antioxidants reached to approximately 8 billion dollars in 2002. This figure is anticipated to escalate to 11.7 billion dollars by 2029, indicating a promising compound annual growth rate (CAGR) of 5 %. The Asia‐Pacific region currently holds the largest market share, accounting for nearly 50 %, with North America in close pursuit at approximately 21 %.[^4^, ^5^]

Antioxidant can be categorized into two distinct classes: primary and secondary antioxidants, based on their distinct mechanisms of action.^[6]^ Both classes of antioxidants are capable of enhancing the polymer materials service life and reliability by protecting it from oxidative degradation. Primary antioxidants encompass hindered phenols and hindered amines.^[7]^ Secondary antioxidants comprise two supplementary types^[8]^: phosphorus‐containing antioxidants and sulfur‐containing antioxidants.^[9]^ However, the incorporation of primary antioxidants to polymer materials can result in the formation of hydroperoxides, which can generate new free radicals upon exposure to light or heat, potentially accelerating the aging process of polymers.^[10]^ Nevertheless, primary antioxidants typically have a low molecular weight (generally 300–1000), which makes them highly volatile and prone to migration to the material's surface and subsequent diffusion from the polymer matrix, particularly during processing or use. This can lead to the depletion of the additive and a concomitant reduction in the material's oxidative performance.

Phosphite antioxidants, a category of highly effective phosphorus‐based antioxidants, are extensively recognized for their efficacy in safeguarding polymer materials during processing, storage, and usage. Their paramount importance is attributed to their capacity to decelerate the aging process of polymers, consequently prolonging their service life. Furthermore, the incorporation of phosphite antioxidants is known to enhance additional properties of the host material. For example, in the field of melt processing industry, t these antioxidants augment thermal stability^[11]^ and melt flowability,^[12]^ which are instrumental in facilitating extrusion molding^[13]^ and preventing the thermal degradation of raw materials. In the realm of textile materials, they are pivotal for maintaining enduring elasticity and color retention.^[14]^ When products are subjected to environmental stressors such as light, heat, moisture, and oxygen, these antioxidants are required to provide enhanced resistance to UV radiation, hydrolysis, and thermal degradation.[^15^, ^16^] The extensive adoption of phosphite antioxidants across various industries has precipitated a substantial expansion in their application scope. They are extensively employed in a variety of materials, including polymers for medical devices, battery components, optical materials, and turbine oils due to their superior performance,^[17]^ which underscores their broad application potential across diverse sectors.^[18]^

**Scheme 1:**
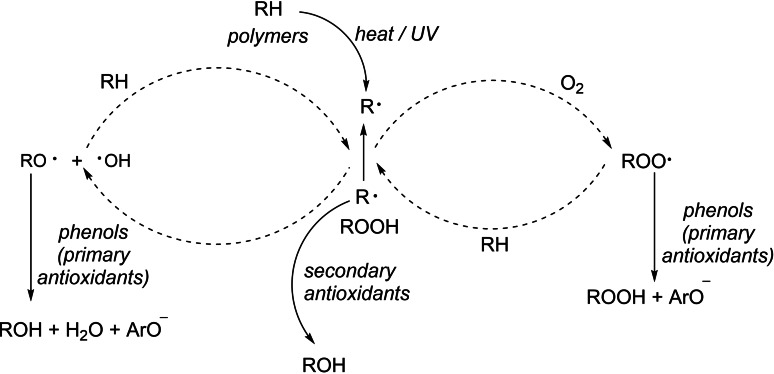
General antioxidation process.

The general mechanism through which primary and secondary antioxidants exert their protective effects is succinctly depicted in Scheme 1.^[19]^ When employed in conjunction with primary antioxidants, such as sterically hindered phenols, phosphite antioxidants act as secondary antioxidants to reduce hydroperoxides generated by the primary antioxidants during their interaction with polymers.^[20]^ Concurrently, as they are oxidized to phosphates, they fulfill a dual role by decomposing hydroperoxides and quenching free radical chains.^[21]^ Furthermore, in contrast to other secondary antioxidants, such as those containing sulfur, phosphite antioxidants provide a diverse array of options for the strategic modification of functional groups. Research indicates that this elevated molecular weight of phosphite antioxidants is pivotal for enhancing the stability, resistance to hydrolysis, and thermal resistance of materials. Therefore, the incorporation of phosphite antioxidants is essential for the modification and improvement of polymer materials.

## 2 Synthesis of Phosphite Antioxidants

In recent years, there has been a notable surge in the synthesis and application of phosphite antioxidants, attributable to their exceptional performance. This advancement has facilitated the successful industrial production and market introduction of several phosphite antioxidant products. To provide a thorough overview of this domain, this review systematically summarizes the current synthesis methods of commercially available phosphite antioxidants. It aspires to be a valuable resource for researchers in the field of antioxidant chemistry, assisting them in the development of novel antioxidant formulations and in the exploration of environmentally benign synthesis strategies that meet the increasingly stringent regulatory requirements for industrial production. This review categorizes the various synthesis methods and precursor materials into three groups: 1) synthesis from phosphorus chloride; 2) synthesis based on transesterification; 3) synthesis from white phosphorus.

### 2.1 Synthesis from Phosphorus Trichloride

Phosphorus trichloride (PCl₃) is characterized by its electron‐deficient nature, a result of the high electronegativity of chlorine atoms. This electron deficiency enables PCl₃ to readily engage in reactions with electron‐rich substances, particularly through nucleophilic substitution involving nucleophiles such as alcohols. Through these reactions, phosphorus trichloride facilitates the synthesis of phosphites. Importantly, PCl₃ serves as a vital reactant in the production of phosphite antioxidants, including antioxidant 168 and antioxidant PEPQ, and other phosphite antioxidants, which are extensively utilized across diverse industries due to their beneficial properties.

#### 2.1.1 Irgafos 168

Irgafos 168, chemically known as tris(2,4‐di‐*tert*‐butylphenyl)phosphite, is a prevalent antioxidant within the phosphite antioxidant category. This compound exhibits superior performance, notably by inhibiting the formation of halogenated hydrocarbons during processing, thereby mitigating mold corrosion. Additionally, it is capable of decomposing hydrogen peroxide generated during the degradation of polymers. When incorporated into the thermal processing of food‐grade plastics, Irgafos 168 adheres to stringent food contact safety standards.^[22]^ Consequently, the synthesis and practical application of Irgafos 168 have garnered significant interest within the scientific community, leading to extensive research aimed at exploring its potential and optimizing its use in various industrial processes.

The synthesis of Irgafos 168 has notably advanced with the elucidation of its reaction mechanism and synthesis methods. Traditionally, this compound was synthesized from 2,4‐di‐*tert*‐butylphenol and PCl_3_ under catalytic conditions (Equation 1). However, due to the steric hindrance of the phenol reactant, the reaction often leads to the formation of by‐products such as dichloro(2,4‐di‐*tert*‐butylphenoxy)phosphane, bis(2,4‐di‐*tert*‐butylphenyl) phosphorochloridite, and hydrogen chloride when interacting with polyhalides, thereby decreasing the reaction efficiency. Consequently, there is a pressing need to develop synthesis methods that yield higher purity and efficiency for Irgafos 168.


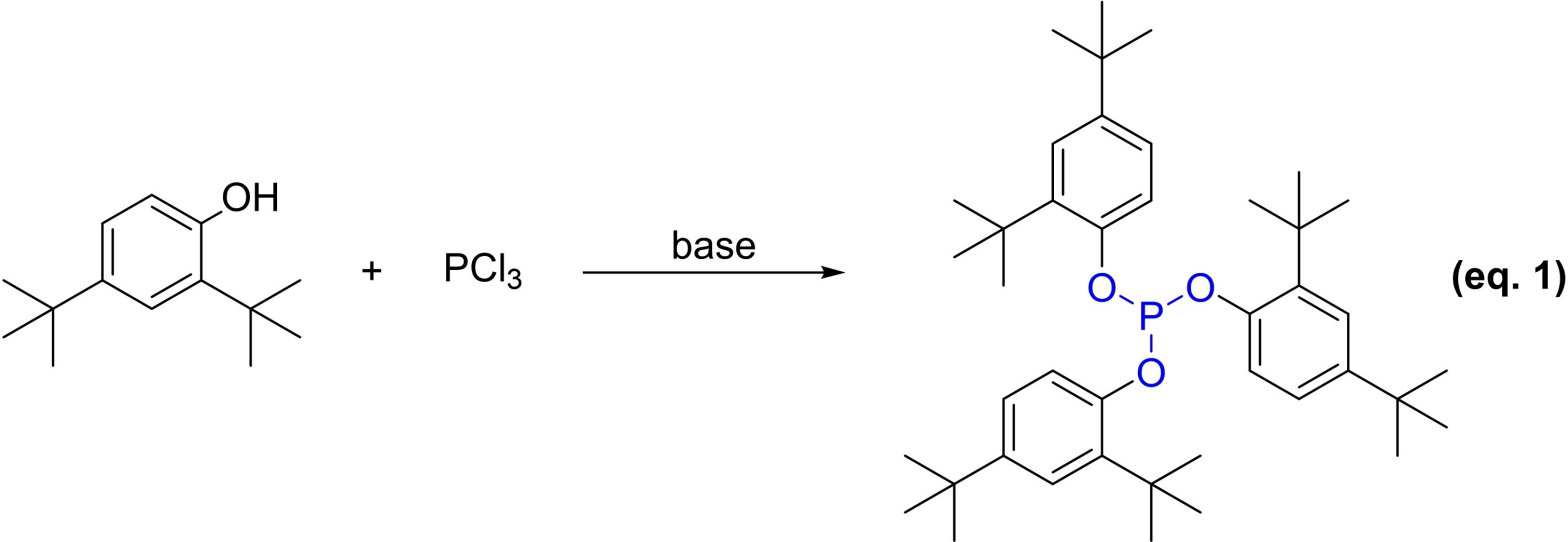


In 1979, the Ciba Guigy Company conducted a study to compare various catalysts in the synthesis of Irgafos 168, using xylene as the solvent. The catalysts examined included amines, ammonium salts, carboxylic acids, guanidine, amides, sulfones, and phosphines. Among these, amine catalysts, particularly dimethylformamide, demonstrated the most effective synthesis performance, achieving a product yield of 85.4 %.^[23]^

In 1981, Hoechst Corporation conducted a comprehensive analysis to assess the influence of solvents on reaction efficiency, from which they inferred plausible correlations between solvent properties and reaction efficiency. The study revealed that solvents significantly enhance the reaction dynamics when the reactant is in a solid state or when the product is a crystalline substance with a high melting point. Organic solvents, including dialkyl ethers, tetrahydrofuran, aromatic hydrocarbons, heptane, and halogenated hydrocarbons, were identified as particularly effective. A notable increase in product yield, reaching up to 90 %, was achieved by employing a higher volume of xylene as the solvent and incorporating a pyridine catalyst. This method, which involves dissolving the reactants in the solvent and directly using the reaction mixture for subsequent processes, streamlines the workflow by eliminating the need for extensive purification steps, thereby reducing costs. Maintaining the reaction temperature between 130–150 °C minimizes the risk of excessive pyrolysis of reactants and products. Moreover, any residual halogenated compounds can be efficiently removed through a mild vacuum treatment, which not only improves the overall yield but also aids in the purification of the product.^[24]^

In the same year, Bayer conducted research experiments on solvents, comparing them with organic solvents, such as benzene, toluene, xylene, and halogenated hydrocarbons (chlorobenzene, methylene chloride, and 1,2‐dichloroethane). Toluene, chlorobenzene, and methylene chloride were found to be preferred choices for use. To improve the reaction process, a saturated NaOH solution was added to increase the volume of aqueous phase and adjust the pH value. This adjustment effectively promotes the reaction. The presence of solvents and diluents aids in separating organic and inorganic phases, facilitating the removal of phosphoacyl impurities using relatively simple methods, thereby enhancing the quality of the final product.^[25]^

In 1991, Uniroyal Chemical Company selected hexane as the solvent to ensure complete dissolution of the reactants during the reaction process. The reaction process was further refined by precisely controlling the temperature to facilitate crystallization of the product, which was subsequently recrystallized using isopropanol. A significant yield of 94 % was achieved by employing 2‐mercaptobenzothiazole as a catalyst, with an optimal dosage range of 0.005–10 mol %. This method is notable for its high reaction efficiency and the potential for catalyst reusability, making it an effective approach for the industrial‐scale production of antioxidant 168.^[26]^

#### 2.1.2 Antioxidant 626

Currently, antioxidant 626 is widely use in various polymer materials, such as polyethylene (PE), polypropylene (PP), polystyrene (PS), polyamide, polycarbonate, and acrylonitrile butadiene styrene (ABS), especially when used combined with phenolic antioxidants. Although it can be utilized as an alternative to antioxidant 168,^[27]^ it is important to note that its resistance to hydrolysis is inferior, necessitating careful consideration of its moisture and heat resistance. The phosphorus trichloride route involves the reaction of phosphorus trichloride with pentaerythritol to form an intermediate of 3,9‐dichloro‐2,4,8,10‐tetraoxa‐3,9‐diphosphaspiro[5.5]undecane, which subsequently reacts with 2,4‐di‐*tert*‐butylphenol to produce the antioxidant 626 (Equation 2).


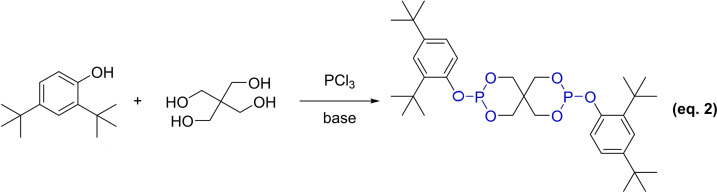


In 2013, Manju's group disclosed a one‐pot method to synthesize antioxidant 626. Initially, they added triethylamine and phosphorus trichloride to a pre‐cooled toluene solution of pentaerythritol, followed by continuous stirring for 1 hour. Subsequently, a toluene solution of 2,4‐di‐*tert*‐butylphenol was introduced to the mixture, which was then refluxed for 5 hours and stirred at room temperature overnight. The reaction solution was filtered, the solvent was evaporated, and the product was isolated to yield antioxidant 626. This innovative approach eliminates the need for the recovery and quenching of excess amount of PCl_3_, thereby reducing the recovery costs and ensuring a complete reaction.^[28]^

In 2023, Duan's team reported a novel method for synthesizing antioxidant 626. Their procedure comprised the following sequential steps: (1) addition of xylene, pentaerythritol, and triethanolamine to the reaction vessel, followed by thoroughly stirring to form the initial mixture; (2) introduction of phosphorus trichloride to the initial mixture to generate an intermediate; (3) incorporation of 2,4‐di‐*tert*‐butylphenol into the intermediate‐containing material to produce a solution containing antioxidant 626; and (4) separation of the auxiliary antioxidant 626 from the mixture. This method is characterized by its environmental friendliness, producing the desired product with low impurities and high yield.^[29]^

#### 2.1.3 Antioxidant PEPQ

Developed by Sandoz company in Switzerland, antioxidant PEPQ, chemically designated as tetra(2,4‐di‐tert‐butylphenol)‐4,4′‐biphenyl diphenyl phosphite, is an efficient phosphorus‐based secondary antioxidant marketed under the trade name Sandostab P‐EPQ. With a molecular weight up to 1035, PEPQ features rare P−O and C−P bonds. The polymer compound exhibits exceptional performance in hydrolysis stability and resistance to high‐temperature oxidation with a melting point of 185–186 °C.

Despite its effectiveness, the commercial product Sandostab P‐EPQ is not a pure crystalline substance, as it contains only 70 % of the active ingredients and encounters significant technical challenges. The reactants implicated in the synthesis of P‐EPQ can be deduced from its molecular structure, which includes biphenyl, 2,6‐di‐*tert*‐butylphenol, and phosphorus trichloride (PCl_3_). Consequently, to augment the efficiency of PEPQ synthesis, further research breakthroughs are imperative to uncover more effective synthetic methodologies.

**Scheme 2:**
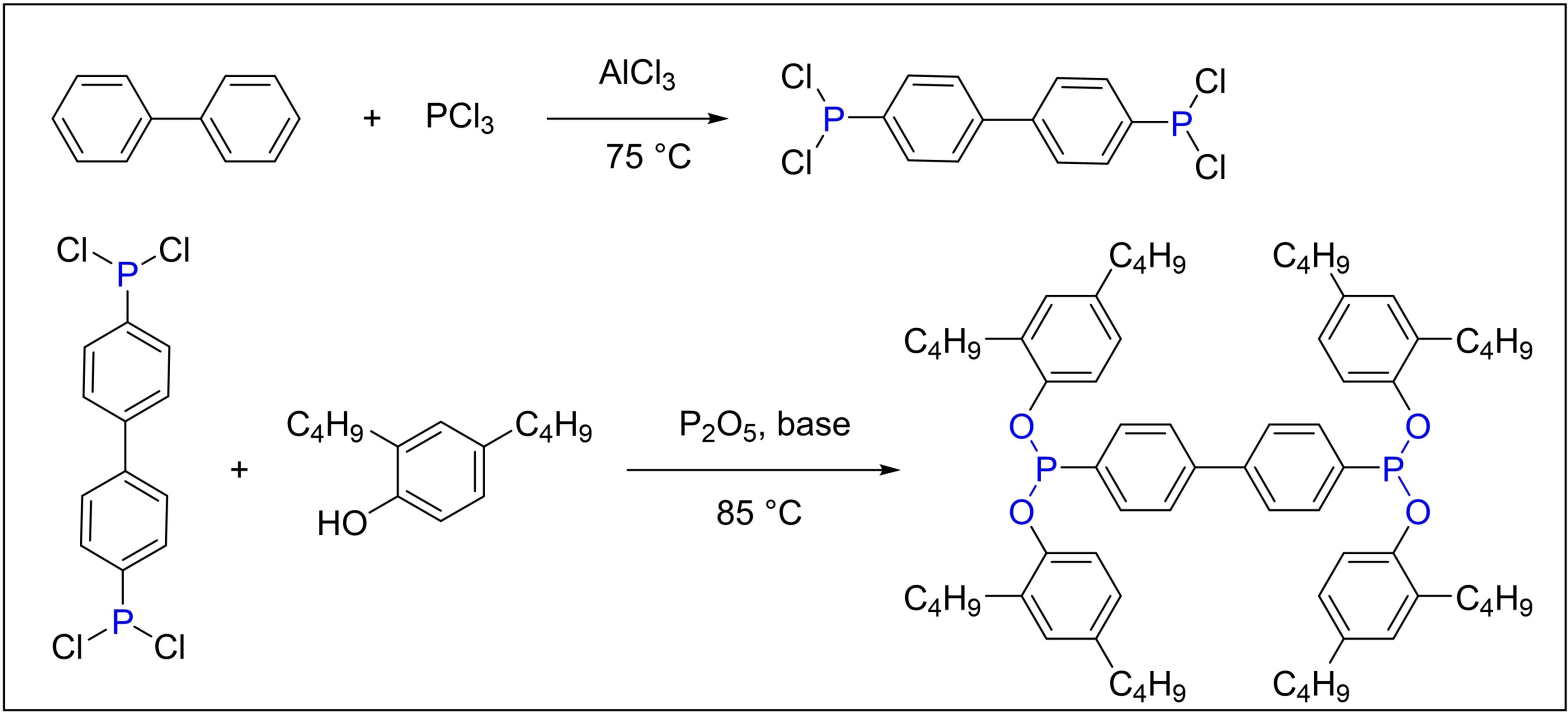
The synthesis of antioxidant PEPQ with PCl_3_.

In 1997, Great Lakes Chemical, an Italian corporation, opted for aluminum trichloride as a catalyst to facilitate the reaction by forming complexes with the reactants (Scheme 2). The separation of the complexed solid aluminum trichloride and its byproduct was achieved through conventional centrifugation or filtration, thereby streamlining the process and reducing associated costs. After completing the reaction, any unreacted PCl_3_, along with the solvents chlorobenzene and toluene, were removed via vacuum distillation to prevent impurity contamination of the product. The process temperature was meticulously maintained below 10 °C to induce the formation of a novel crystalline form of tetrakis‐(2,4‐di‐*tert*‐butylphenol)‐4,4′‐biphenylene diphosphate with a purity of 98 %. Propylene ketone was employed as the crystallization medium in this method, which presents several benefits, including facile product separation, diminished production expenses, and enhanced product purity.^[30]^

In 2007, Wang and colleagues refined the synthesis conditions for the antioxidant PEPQ through a series of controlled experiments. The authors commenced the synthesis process by adjusting the stoichiometric ratio of the reactants to n (biphenyl): n (PCl_3_)=1 : 6, facilitating the formation of the intermediate 4,4′‐biphenylphosphine dichloride under the catalytic influence of AlCl_3_. The elevated PCl_3_ ratio was necessitated by the requirement for additional PCl_3_, as some of it existed in the vapor phase during the reflux process. The intermediate was then reacted with 2,4‐di‐*tert*‐butylphenol, utilizing benzene as the solvent. Triethylamine was employed to neutralize the hydrogen chloride gas produced, and the product was subsequently subjected to recrystallization using methyl ethyl ketone. The molar ratio of n (2,4‐di‐*tert*‐butylphenol): n (4,4′‐biphenylphosphine dichloride): n (triethylamine) was optimized to 4.4 : 1 : 4.8. Despite these meticulous optimization efforts, the process resulted in a yield of only 57.2 % of the target product, underscoring the scope for substantial enhancement in future research aimed at optimizing this synthesis protocol.^[31]^

The experimental evidence shows that PEPQ synergistically interacts with traditional phenolic antioxidants to effectively counteract oxidative stress. Furthermore, PEPQ has been found to significantly amplify the antioxidant efficacy of the host materials it is paired with. This synergistic enhancement suggests promising avenues for innovation in antioxidant technology, potentially leading to the creation of more robust and efficient antioxidant formulations.^[32]^

#### 2.1.4 Antioxidant PEP‐36

Antioxidant PEP‐36, commercially known as Mark PEP‐36, which is bis(2,6‐di‐*tert*‐butyl‐4‐tolyl) pentaerythritol phosphite, featured by a unique spirocyclic structure of alcohol bisphosphite. The antioxidant PEP‐36, distinguished by its high steric hindrance, elevated molecular weight, and substantial effective phosphorus content, exhibits several desirable properties such as high hydrolysis stability, low volatility, and excellent processing stability. Unlike the traditional phosphorus‐containing antioxidant 168, which tends to yellow at elevated temperatures, PEP‐36 maintains its color and performance. Its stability is on par with the well‐established Ultranox 626, yet it surpasses it in terms of hydrolytic stability.

**Scheme 3:**
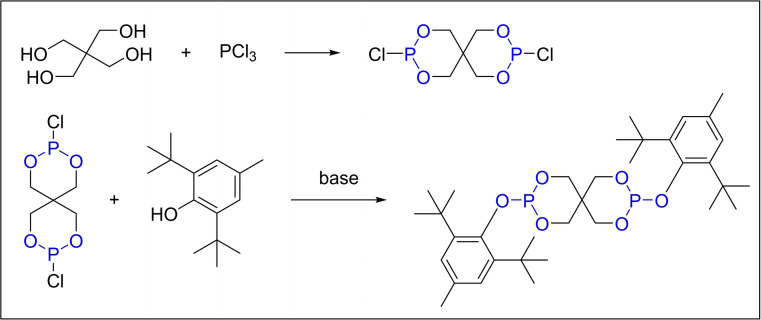
Synthesis of antioxidant PEP‐36 with PCl_3_.

The synthesis of PEP‐36 follows a general protocol consisting of two primary steps. Initially, the intermediate dichlorodiphosphite pentaerythritol was obtained by utilizing PCl_3_ along with pentaerythritol as the starting materials. Subsequently, the intermediate was subjected to a substitution reaction with 2,6‐di‐*tert*‐butyl‐4‐methylphenol to produce the final product PEP‐36 (Scheme 3). However, the substitution reaction in the second step faces challenges due to the significant steric hindrance from the 2,6‐di‐*tert*‐butyl groups on the aryl motif. Consequently, separating product impurities becomes complex.

In 1989, Hobbs and colleagues utilized tri‐*n*‐alkylamine as the catalyst in the synthesis, a method that gained widespread acceptance in the subsequent production stages. However, challenges related to separation difficulty and significant recovery costs have hindered the practical application of this type of catalyst in large‐scale production and industrial environments, despite the mild reaction conditions it provides.^[33]^

**Scheme 4:**
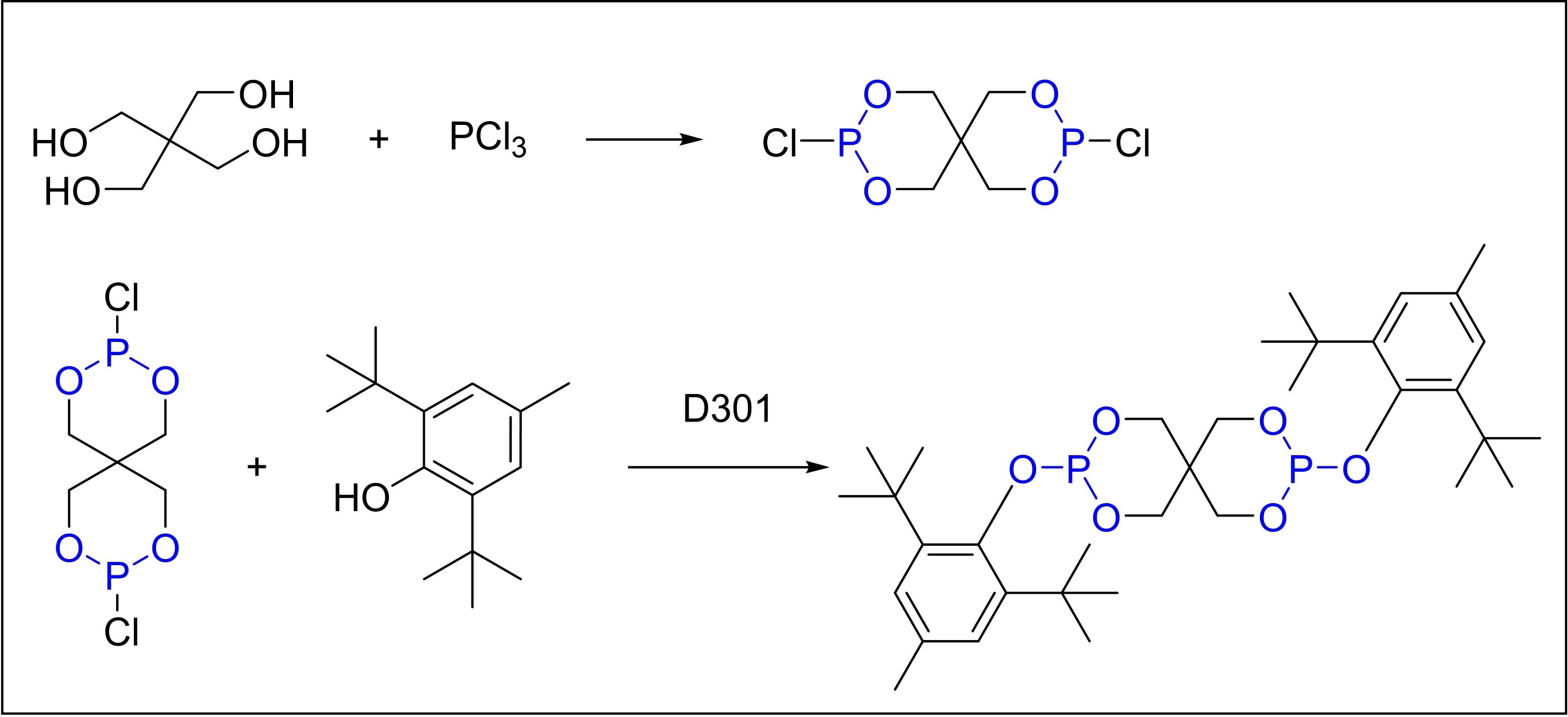
Synthesis of antioxidant PEP‐36 with PCl_3_ using D301 as the catalyst.

In 2005, He and colleagues employed a one‐pot synthesis strategy in a toluene medium under nitrogen protection to produce PEP‐36 (Scheme 4). Traditional amine catalysts, commonly utilized in PEP‐36 synthesis, offer mild reaction conditions but are challenging to recover and separate, thereby increasing production costs and hindering industrial scalability. The He research team utilized ion exchange resins as catalysts, which are easier to recover and separate. To address these challenges, He's team innovatively applied ion exchange resins as catalysts, simplifying the recovery and separation processes. Among the five commercially available ion exchange resins tested‐D301, D370, D371, D315, and D290–D301, a large‐pored weakly basic styrene anion exchange resin, demonstrated superior catalytic activity.

This catalyst not only activates the P−Cl bond in PCl_3_, promoting the forward reaction, but also sequesters the HCl gas generated during the reaction, thereby enhancing reaction efficiency and reducing the alkali requirement for gas absorption. The resultant yield was 67.5 %, suggesting room for improvement in production efficiency. Compared to traditional amine catalysts, the ion exchange resin catalyst presents distinct advantages in separation and recovery ease, making it a more viable option for industrial applications. The adoption of D301 has the potential to streamline industrial production processes and minimize environmental pollution associated with waste alkali disposal.^[34]^

In 2015, Wu and colleagues introduced an innovative one‐pot method for synthesizing PEP‐36, utilizing triethylamine as both an organic base and acid binding agent to regulate the pH.^[35]^ Diatomaceous earth was strategically employed as an adsorbent within this process. By implementing this method, impurities like salt, which tend to adhere to the product, could be easily removed through a straightforward filtration process. Consequently, the purity of the final product was elevated to 99.07 %. Additionally, this approach effectively mitigated the problem of excessive by‐products and impurities arising from significant steric hindrance, thereby reducing production costs compared with the use of high‐consumption, costly ion exchange resins.

In 2020, building on the research foundation established by Wu *et al*.,^[35]^ Shanghai Petrochemical Xinier Company developed a protocol for synthesizing the antioxidant PEP‐36. The process involved using trimethylbenzene as the solvent and diatomaceous earth as a filter aid. To adjust the pH value and facilitate dissolution, a mixture of NaOH, triethylamine, and dibutylamine was incorporated. The protocol achieved a yield of 80.7 %, indicating that manipulating the solvent ratio or precisely controlling the pH value can significantly enhance the reaction's progress.^[36]^

In 2022, Zhejiang Wansheng Company in China developed a novel method for the industrial‐scale synthesis of PEP‐36. The process commenced with a reaction between pentaerythritol and phosphorus trichloride, catalyzed by tripropylamine. Subsequently, BHT (butylated hydroxytoluene) was introduced to the reaction mixture, accompanied by a dropwise addition of a blend of tripropylamine and tributylamine, which served as binding agents. By refining the type of acid binder and the solvent, as well as meticulously adjusting and controlling the conditions in the second reaction step, significant enhancements in yield, reaction time reduction, and product purity were realized. This innovative approach represents a milestone in the production process.^[37]^

#### 2.1.5 Antioxidant 9228

Antioxidant 9228, with a molecular weight of 852, is chemically defined as bis(2,4‐di‐*tert*‐butylphenyl)pentaerythritol diphosphite. Engineered as a second‐generation phosphite antioxidant to address the inadequate hydrolysis resistance of antioxidant 626, antioxidant 9228 demonstrates superior thermal stability and an expedited decomposition rate of hydrogen peroxide. The distinctive molecular architecture of antioxidant 9228 endows it with enhanced hydrophobic characteristics within its matrix, subsequently bolstering its hydrolytic resistance. Given its exceptional thermal stability and swift decomposition of hydrogen peroxide, antioxidant 9228 has earned recognition as the preeminent heat‐resistant phosphite antioxidant on the global stage. It sustains remarkable thermal stability at temperatures surpassing 300 °C, an attribute largely attributed to its high molecular weight and distinctive molecular architecture. This remarkable heat resistance and thermal stability renders antioxidant 9228 highly effective in polymer applications at high processing temperatures, such as polyarylenesulfidesulfone (PASS), polyethylene terephthalate (PET), polybutylene terephthalate (PBT), polycarbonate (PC), and polyphenylene sulfide (PPS).[^10^, ^12^, ^38^]

**Scheme 5:**
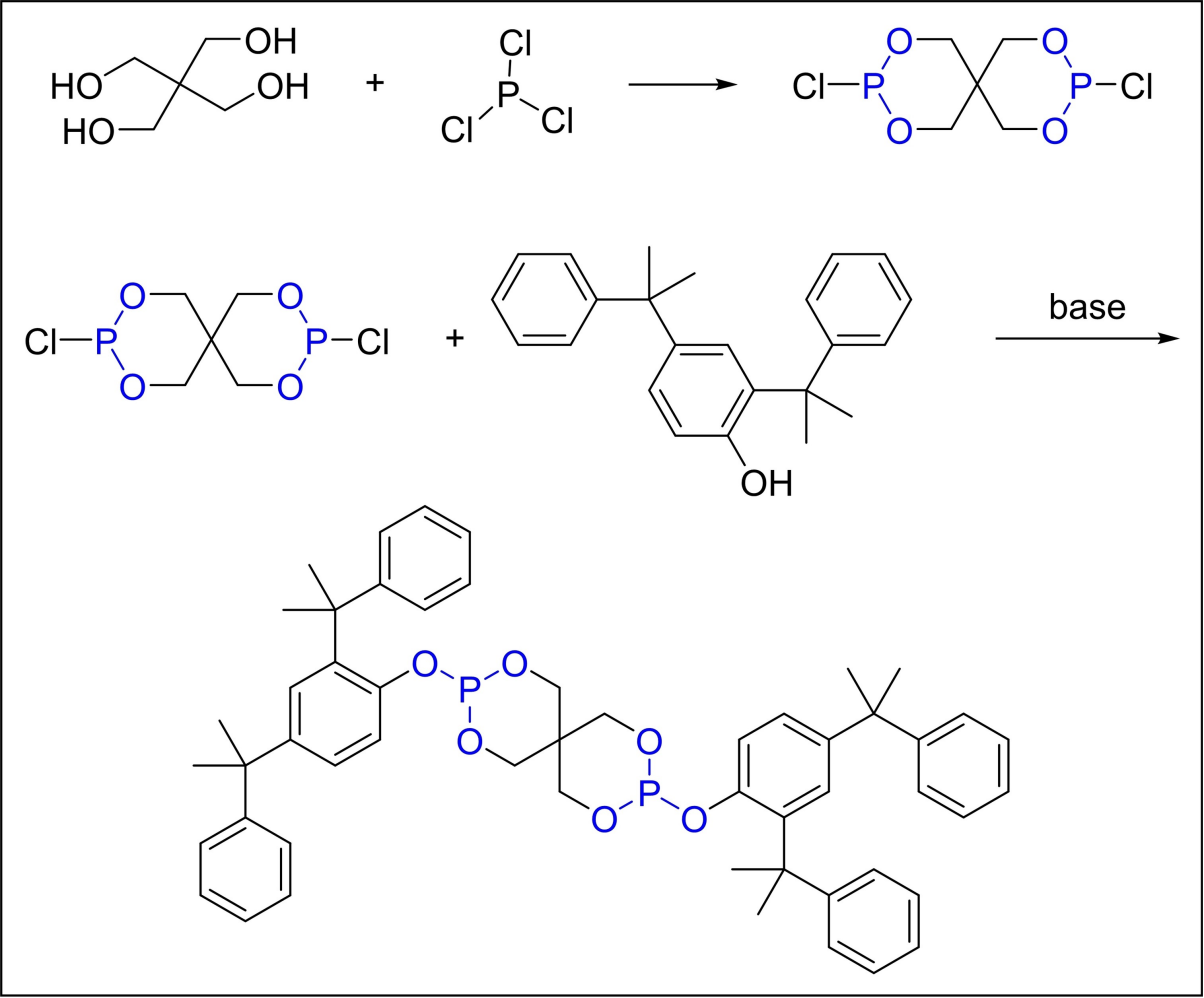
Synthesis of antioxidant 9228 with PCl_3._

In 2011, Donald Stevenson *et al*. introduced a novel method to synthesize antioxidant 9228 by directly esterifying, 2,4‐dicumylphenol with PCl_3_. This approach used these compounds as both reactants and solvents in a reaction vessel pre‐charged with pentaerythritol (Scheme 5). The choice of phase‐transfer catalyst, such as benzyltriethylammonium bromide, hexadecyltrimethylammonium bromide or chloride, tetraethylammonium bromide or chloride, tetramethylammonium bromide or chloride, tetraethylammonium bromide or chloride, was crucial to the success of this synthesis. Experimental results indicated that benzyltriethylammonium bromide emerged as the most effective catalyst, with the optimal catalyst usage falling within the range of 3.0–5.0 % by weight relative to pentaerythritol. As a result, the yield of the reaction reached an impressive 92 %. This successful synthesis process not only enabled the prevention of the degradation of host polymer but also elevated the inherent quality of the final products, offering enhanced thermal and photo‐degradation resistance for their intended applications.^[39]^

In 2013, Dai's group investigated an alternative method to synthesize antioxidant 9228, utilizing phosphorus trichloride as a raw material. This approach was developed to address several challenges associated with transesterification reactions, including high raw material consumption, excessive by‐product formation, elevated energy consumption, and increased production costs. By focusing on the availability and cost of raw materials, the method aimed to mitigate these issues effectively. The synthesis of the antioxidant 9228 commenced with the gradual addition of a toluene solution of 2,4‐dicumylphenol to a toluene solution of phosphorus trichloride. The presence of a single reactive functional group in the molecular structure of 2,4‐dimethylphenol effectively curtailed undesired side reactions, thereby enhancing the purity and yield of the intermediate products. Following completion of this reaction, the intermediate was not isolated; instead, pentaerythritol was directly introduced to the existing reaction mixture. Following post‐treatments, the desired antioxidant 9228 was successfully produced.

The molar ratio of the raw materials was refined to obtain the desired product, with the optimal ratio being 1.0 : 2.18 : 2.0 for n(pentaerythritol) : n (PCl_3_) : n (2,4‐dicumylphenol). This method, defined by its stable and manageable reaction conditions, was found to be operationally straightforward, thereby ensuring product quality. An excess of phosphorus trichloride was employed to ensure complete reaction conversion. A variety of catalysts, including dimethylformamide, triethylamine, pyridine, and organic ammonium salts, were evaluated for their catalytic efficacy. The experimental findings indicated that organic ammonium salts exhibited superior catalytic efficiency. It is recommended to mix 1.05 % by mass of these salts with 2,4‐dimethylphenol. Toluene served as the reaction solvent, with isopropanol utilized as the crystallization solvent, culminating in a notably high product yield of 78 %.^[40]^

#### 2.1.6 Adk Stab HP‐10

Adk Stab HP‐10, chemically designated as 2,2′‐methylenediphenol‐bis(2‐ethylhexylphosphate), represents a novel antioxidant engineered to counteract the varying susceptibilities to hydrolysis commonly observed in traditional organophosphates. This advanced molecule boasts a unique structural configuration, wherein the phosphorus atom is covalently bonded to a bulky hindered phenol moiety exhibiting pronounced steric hindrance at the *ortho* position. This strategic molecular arrangement endows HP‐10 with the formation of a hydrophobic eight‐membered ring, which significantly enhances its resistance to hydrolysis. This synergistic combination results in an antioxidant with unparalleled resistance to hydrolysis, marking a significant advancement in the field of polymer stabilization.

**Scheme 6:**
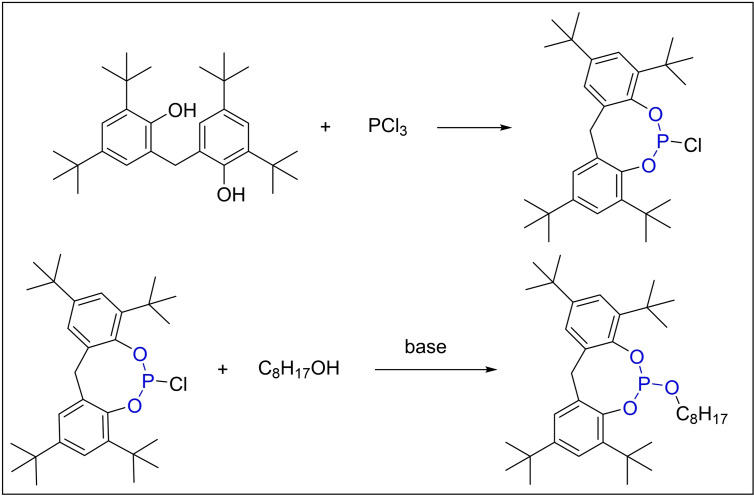
The synthesis of Adk Stab HP‐10 with PCl_3_.

In 2012, Xu and colleagues executed a two‐step esterification process within a single reactor, utilizing 2,2′‐oxybis(4,6‐di‐*tert*‐butylphenol), isooctanol, and phosphorus trichloride as the primary feedstocks. They employed an organic base composite as a catalyst and petroleum ether as the solvent (Scheme 6). The initial esterification stage, enhanced by the composite organic base catalyst, led to a more thorough reaction. In the subsequent phase, triethylamine acted as a proton scavenger, effectively neutralizing the HCl gas produced. This process converted the gas into triethylamine hydrochloride, a solid salt that remained dispersed within the reaction mixture. The implementation of straightforward filtration procedures, coupled with the meticulous regulation of the scavenger concentration to sustain a marginally alkaline environment, significantly curtailed the proliferation of by‐products. This strategic approach enhanced the product yield to a commendable 84.3 %.^[41]^

In 2020, Xin and Wang *et al*. introduced a new continuous‐flow synthesis method for triaryl phosphite and its derivatives, utilizing triethylamine (TEA) as a catalyst (Equation 3).^[42]^ The process, with a 20 second residence time, produced the target compound in a microreactor, greatly reducing reaction time compared to traditional batch methods. Optimized conditions yielded high‐quality products from various substrates. The stoichiometric reactant ratio ensured efficiency, cost‐effectiveness, and eco‐friendliness. A scaled‐up setup enabled the production of up to 18.4 kg/h of tris(2,4‐di‐*tert*‐butylphenyl) phosphite at an 88 % yield, facilitating industrial scale‐up with reduced costs and improved safety.


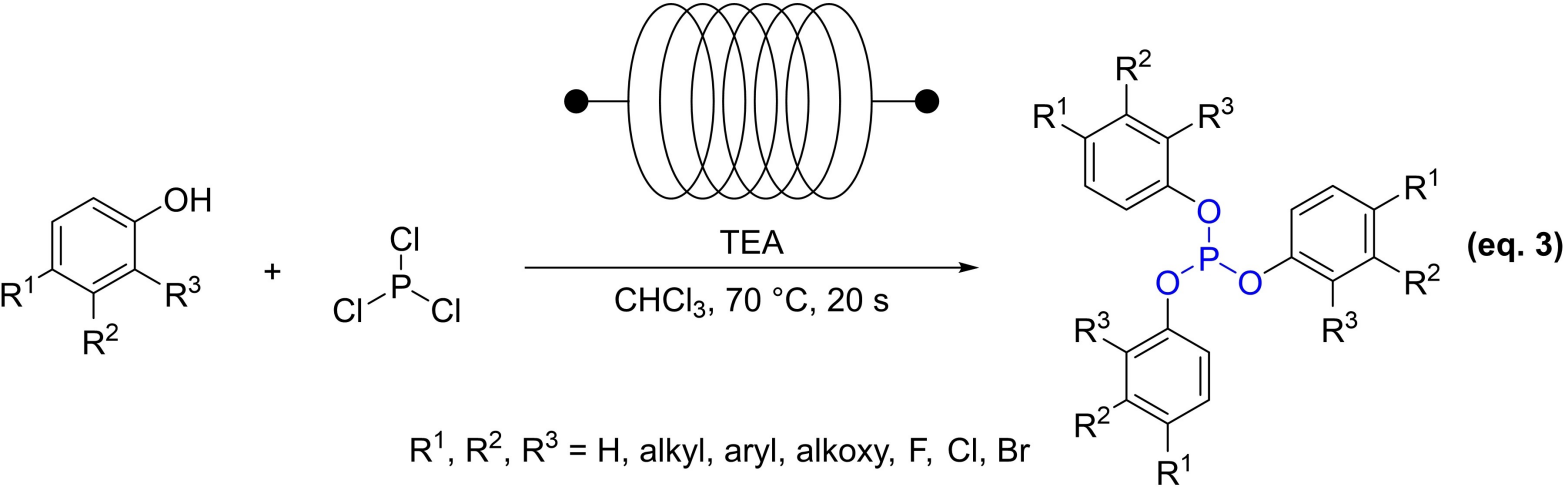


**Scheme 7:**
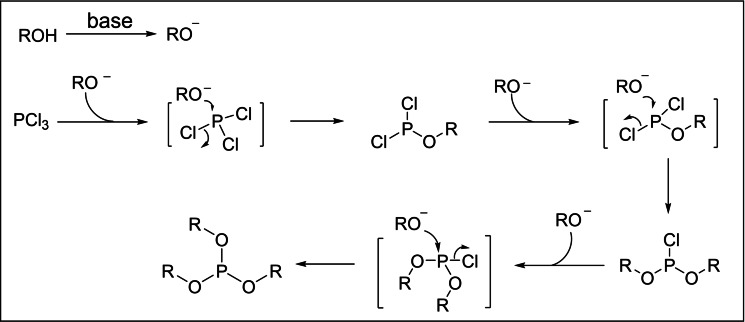
Mechanism for phosphite antioxidants synthesized with phosphorus trichloride.

As shown in Scheme 7, a possible mechanism for the synthesis of phosphite antioxidants with PCl_3_ is proposed.^[42]^ Initially, under the influence of bases, phenols or alcohols lose hydrogen to form ArO^−^ or alkoxy anions. Subsequently, in an environment containing an acid‐binding agent, the anion executes three nucleophilic attacks on the phosphorus atom with phosphorus trichloride. This sequence of reactions induces the departure of the chloride ion, culminating in the establishment of a phosphorus‐oxygen bond and the concomitant displacement of the chloride. As a consequence, the desired phosphite product is synthesized.

### 2.2 Synthesis Based on Transesterification

Transesterification represents a pivotal technique in the realm of organic chemistry, notably for the production of complex ester compounds and has been extensively applied in the synthesis of complex phosphite compounds as well. The versatility of the transesterification process lies in its ability to facilitate the formation of these esters with high efficiency and selectivity, making it an attractive option for both academic and industrial applications. This technique offers a straightforward and facile approach to synthesizing phosphite antioxidants. It is particularly well‐suited for the synthesis of alkyl phosphite esters that possess a carbon chain of three or more atoms, with a prime example being antioxidant 618.

Antioxidant 618, chemically denoted as 3,9‐bis(octadecyloxy)‐2,4,8,10‐tetraoxa‐3,9‐diphosphaspiro[5.5]undecane, is recognized for its exceptional antioxidant properties. However, the presence of residual phenol from the manufacturing process can engender deleterious effects. Such residual phenol has the potential to induce discoloration, notably yellowing, which may restrict the applicability of the antioxidant in certain sensitive applications. This issue underscores the necessity for stringent purification processes to mitigate the phenol content and ensure the product's suitability for a broad spectrum of uses.

**Scheme 8:**
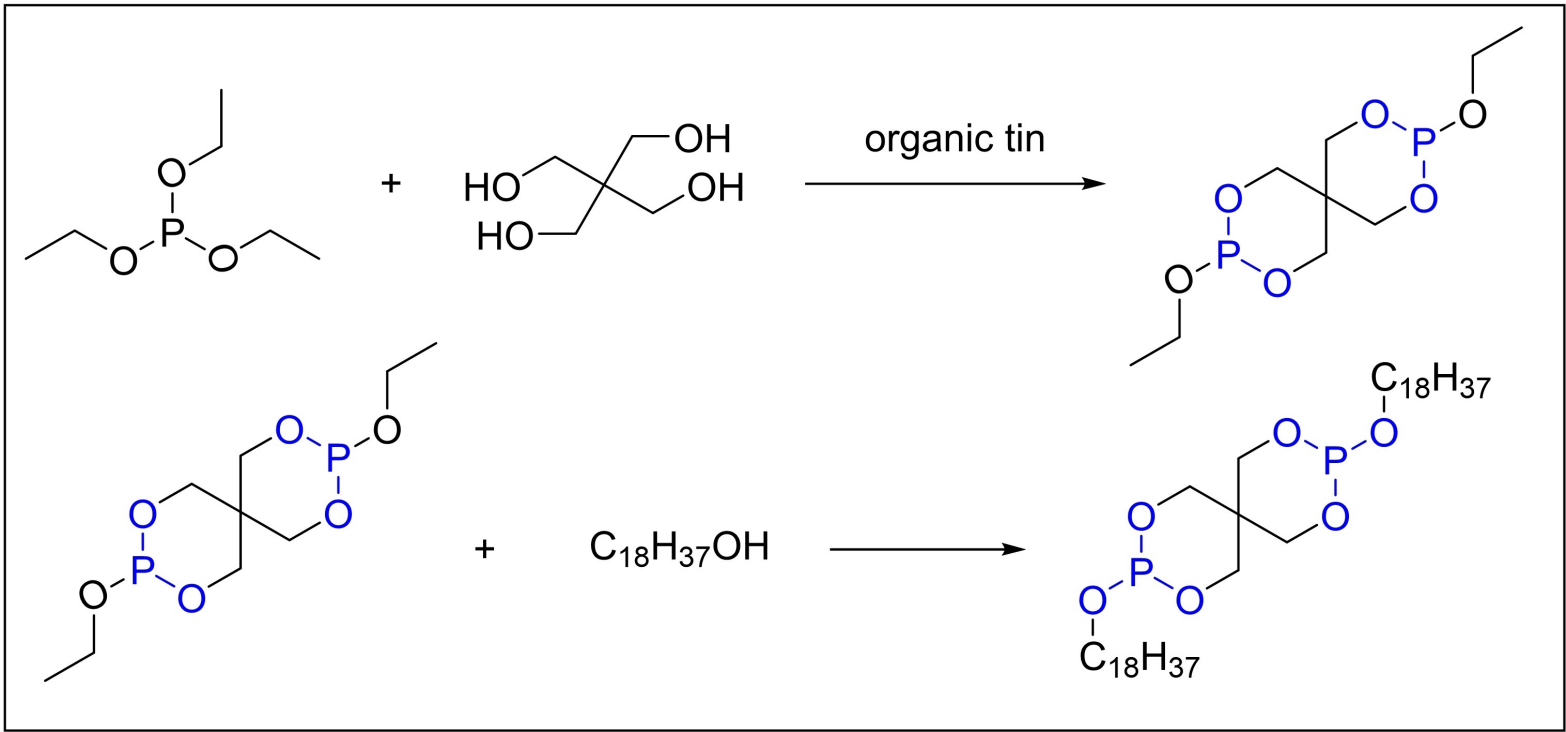
Synthesis of Antioxidant 618 via transesterification catalyzed by organic tin.

In 2006, Pan and colleagues proposed a new process for synthesizing antioxidant 618 through transesterification using triethyl phosphite, pentaerythritol, and octadecanol as raw materials (Scheme 8).^[43]^ The innovative method for obtaining the product, pentaerythritol diphosphite, is characterized by its high yield and simple treatment process. This approach is distinguished by its environmental friendliness and non‐toxicity, as it eliminates the requirement for organic solvents like toluene. In order to improve the yield, Pan *et al*. investigated various factors that influence the reaction. They evaluated different catalysts, such as sodium methoxide, sodium hydroxide, potassium hydroxide, anhydrous potassium carbonate, and organic tin. Notably, organic tin demonstrated superior performance, achieving an impressive yield of 98.5 %. It was found that the best results were achieved when the catalyst dosage was 3 % of the amount of pentaerythritol (mass fraction), along with maintaining a reaction temperature of 130 °C and a reaction time of 2.5 hours. Furthermore, the researchers underscored the paramount significance of adhering to specific molar ratios: 2.05 of triethyl phosphite to pentaerythritol and 2.05 of octadecanol to the intermediate pentaerythritol diphosphite. These precise ratios are essential to guarantee a thorough and complete reaction.

**Scheme 9:**
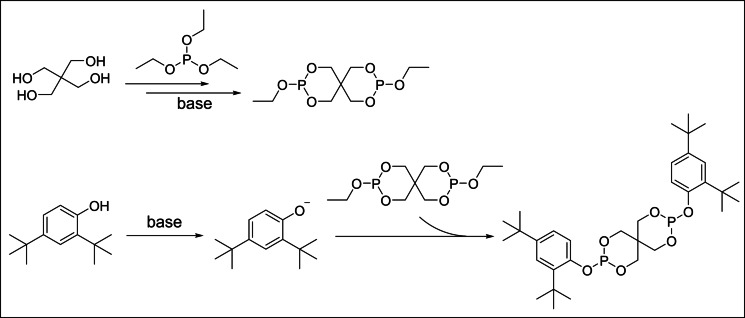
Synthesis mechanism of phosphite antioxidants via transesterification.

A possible mechanism for the synthesis of phosphite antioxidants via transesterification is proposed as shown in Scheme 9.^[43]^ Initially, under alkaline conditions, transesterification proceeds with triethyl phosphite to yield 3,9‐diethoxy‐2,4,8,10‐tetraoxa‐3,9‐diphosphaspiro[5.5]undecane as an intermediate. Subsequently, this intermediate compound undergoes a subsequent transesterification reaction with 2,4‐di‐*tert*‐butylphenol, culminating in the formation of the final product.

### 2.3 Synthesis from White Phosphorus

White phosphorus, a pivotal resource within the phosphorus family, plays a significant role in the chemical industry due to its extensive application in the synthesis of organophosphorus compounds.[^44^, ^45^, ^46^, ^47^] Furthermore, white phosphorus is also employed as a key precursor in the production of phosphite compounds,^[48]^ which are known for their diverse applications in chemical synthesis and as intermediates in the preparation of other phosphorus‐containing molecules. The versatility of white phosphorus in these syntheses underscores its importance in modern chemical research and industrial processes.

Triphenyl phosphite predominantly functions as a secondary antioxidant in the stabilization of polyolefins, polyorganosiloxanes, and epoxy resins, while also fulfilling the dual role of heat and light stabilizer for cellulose esters. Additionally, triphenyl phosphite serves as a pivotal intermediate in organic synthesis, commonly derived from the reaction between phenols and phosphorus trichloride. The volatility of both phenols and phosphorus trichloride during the synthesis can lead to heightened raw material expenses and environmental problem. Furthermore, the production of phosphorus trichloride poses safety hazards and is characterized by suboptimal atom economy and selectivity, frequently culminating in the generation of substantial chlorinated waste byproducts, thereby exacerbating separation costs. Therefore, the development of cleaner phosphorus sources for the synthesis of these compounds holds substantial significance, potentially offering a more environmentally friendly and economically viable approach to chemical production.

In 2005, the Abdreimova group synthesized various esters of phosphoric and hypophosphoric acids directly from white phosphorus and aliphatic (or aromatic) alcohols using CuX_2_ or FeX_3_ (X=Cl, NO_3_, C_3_H_7_CO_2_) as catalysts under aerobic conditions. The presence of the catalyst eliminated the possible side reaction route in which white phosphorus is oxidized to phosphorus oxide by oxygen free radical chain, greatly enhancing the overall efficiency of the reaction (Equation 4).^[49]^


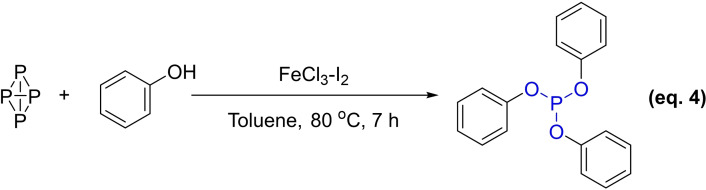


In 2021, Tang *et al*. introduced a novel protocol for synthesizing triaryl phosphites using a base and diphenyl diselenide as co‐catalysts, with white phosphorus (P_4_) and phenols as starting materials, eliminating the need for halogens or transition metals. (Equation 5).^[50]^ Their initial synthesis study utilized P_4_ and *p*‐methoxyphenol as reactants, where a mixture of K_3_PO_4_, and diselenide was heated at 60 °C under argon atomosphere. Phenols containing electron‐donating groups, such as MeO, EtO, BnO or MeS, exhibited high reactivity with P_4_, generating the corresponding products in the yield of 66–95 %. The research teams’ findings showed that it is feasible to synthesize different phosphorus‐containing compounds using white phosphorus as the source, presenting new approaches to producing phosphite antioxidants.


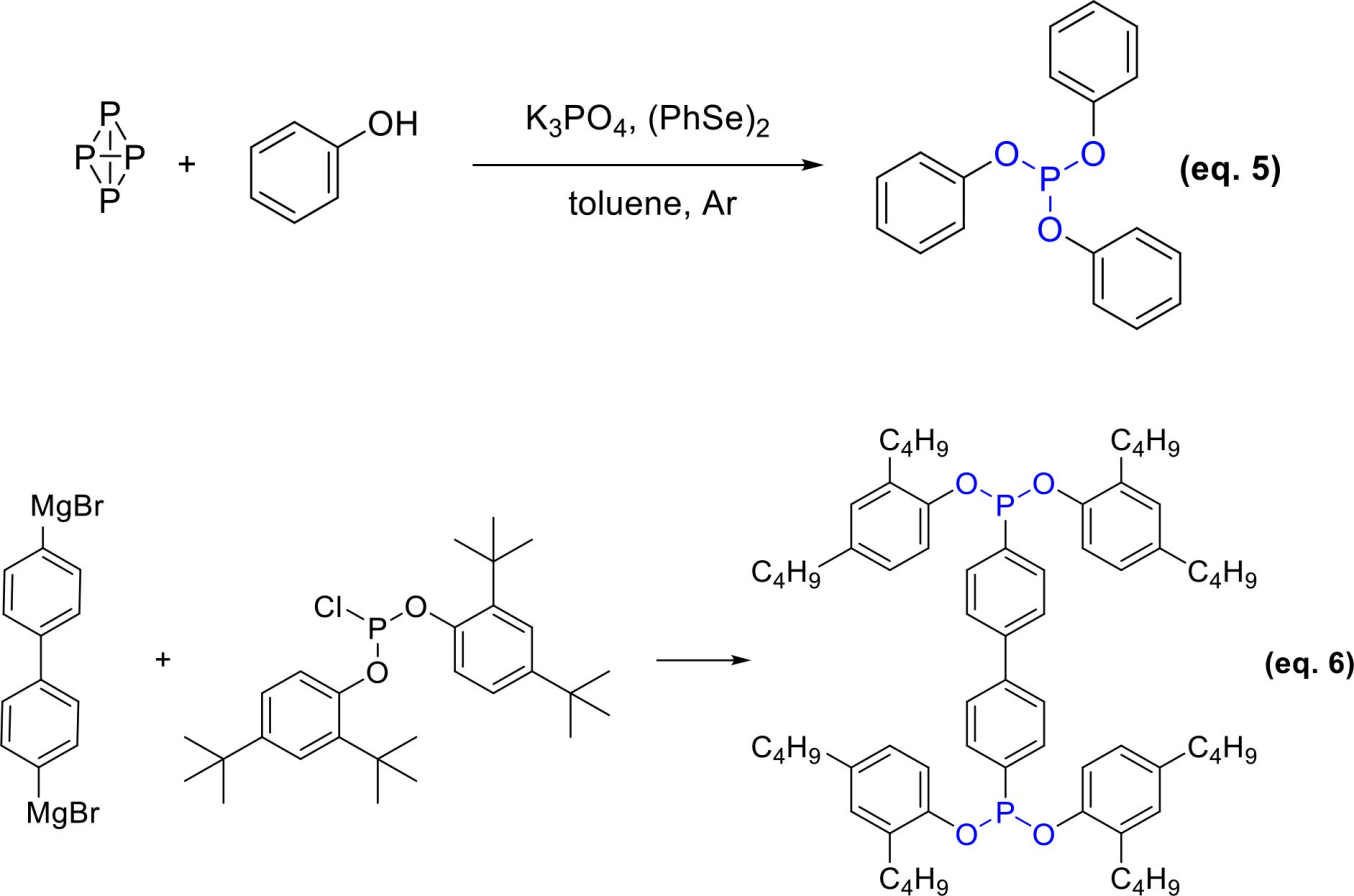


Grignard reagents represent an alternative approach for the synthesis of phosphite antioxidants, complementing the three previously discussed conventional methods. In 1996, a team led by M. Böhlshar refined the method initially proposed by Sandoz for the synthesis of P‐EPQ. This refined process begins with p‐dibromobiphenyl, which reacts with magnesium in an ether solution to form a Grignard reagent. This reagent then undergoes a reaction with bis(2,4‐di‐*tert*‐butylphenyl) phosphorochloridite, yielding antioxidant P‐EPQ (Equation 6). The incorporation of ultrasonication as an assistant tool in this reaction has been shown to enhance the yield of P‐EPQ to a maximum of 85 %. However, the requirement for an anhydrous environment in this synthetic pathway presents a significant challenge, potentially limiting its scalability and practicality for industrial applications.^[51]^

## 3 Potential Risks of Phosphite Antioxidants

Although phosphite antioxidants are renowned for their remarkable attributes, such as extending the lifespan of polymer materials, preventing discoloration due to oxidation, and even enhancing certain characteristics of the materials they are used with, it is crucial to be vigilant about the potential environmental and health risks associated with the use of phosphorus‐containing antioxidants. There is a possibility that these substances could be detrimental to the environment, living organisms, and human health. When employing phosphite antioxidants, it is essential to be aware of the potential for external migration, which can lead to the dispersion of these compounds into the surrounding environment. Research has indicated that organophosphate esters (OPEs), which are the degradation products of organophosphite antioxidants (OPAs), were detected in 85 samples of indoor dust. Notably, over 78 % of these OPEs were traced back to the oxidation of OPAs.^[52]^ While OPEs are considered to have low toxicity, they can still pose a threat to human health through various exposure pathways, such as skin contact, ingestion, or inhalation. The risks are particularly pronounced for individuals who work in environments where these compounds are commonly used.^[53]^

To mitigate these risks, it is imperative to implement stringent safety measures and to continue researching the long‐term effects of OPEs and OPAs on both human health and the environment. This includes developing alternative antioxidants that offer the same benefits without the associated risks, as well as improving the understanding of how to safely manage and dispose of these materials to minimize their impact on the ecosystem and public health.

## 4 Conclusions

This review provides a thorough examination of synthetic methods for phosphite antioxidants, meticulously categorizing three predominant approaches: using phosphorus trichloride, simple phosphites, and white phosphorus as precursors, and alcohols or phenols to synthesize antioxidants through substitution or transesterification. High‐yield synthetic methods typically require large raw material ratios, leading to increased waste and production costs. These methods also face challenges, including the emission of hydrogen chloride and chlorinated gases, the need for alkaline neutralization of waste, and difficulties in separating catalysts from byproducts and other compounds. Moreover, existing phosphite antioxidants pose some environmental and toxicity concerns. Addressing these issues is crucial for future research, especially considering the growing emphasis on sustainable chemistry. In future, there is a pressing need for research and testing of innovative phosphite antioxidants for a variety of applications in polymer materials, with a focus on enhancing their performance and compatibility with other antioxidants. Furthermore, antioxidant research must progress towards the development of greener, safer, and more stable synthetic methodologies that align with the growing human health concerns and the imperative for sustainable practices.

## Conflict of Interests

The authors declare no conflict of interest.

5

## Biographical Information

*Longzhi Zhu, was born in Hunan Province, China, in 1991. He obtained Bachelor of Engineering degree from Jishou University in 2013, and Ph.D. degree at Hunan University in 2019. He pursued postdoctoral research at Shenzhen University and The Hong Kong Polytechnic University, collaborating with Professors Jun Song and Wai‐Yeung Wong, respectively. In 2022, he became a member of Professor Ke‐Wen Tang's research group at Hunan Institute of Science and Technology. Dr. Zhu's research interests currently focus on the C−H bond functionalization and the development of green synthetic methods for the formation of P−C and P−S bonds in organophosphorus chemistry*.

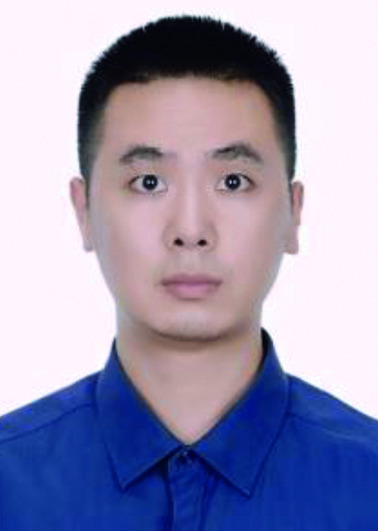

## Biographical Information

*Biquan Xiong, was born in Hunan province, China, in 1987. He obtained Bachelor of Engineering from Jishou University in 2010, and Ph.D. degree from Hunan University in 2015. In 2015, He joined Professor Ke‐Wen Tang's group at Hunan Institute of Science and Technology. He promoted to associate professor since 2020. Dr. Xiong's current research interests focus on the transition‐metal‐catalyzed activation of inert chemical bonds and P−H/P−OH/P−OR bonds, developing green synthetic protocols for the construction of P−C and P−Z bonds in organophosphorus chemistry*.

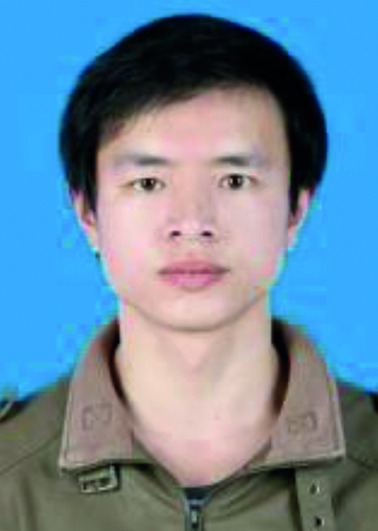

## Data Availability

Data sharing is not applicable to this article as no new data were created or analyzed in this study.
